# A circulating three-miRNA panel (*hsa-miR-29b-3p*, *hsa-miR-19b-3p*, *hsa-miR-30e-5p*) for early-stage ovarian cancer detection: a machine-learning bioinformatics approach

**DOI:** 10.3389/fgene.2026.1827636

**Published:** 2026-08-12

**Authors:** Ahmed El Hosseiny, Meriem Yagoubi, Ahmed Moustafa, Asma Amleh

**Affiliations:** 1 Biotechnology Program, American University in Cairo, New Cairo, Egypt; 2 Department of Biology, American University in Cairo, New Cairo, Egypt

**Keywords:** bioinformatics, biomarkers, early detection, machine learning, microRNA, ovarian cancer, random forest, TCGA

## Abstract

**Background:**

Ovarian cancer (OVCA) remains one of the most lethal gynecological malignancies, primarily due to late-stage diagnosis and the lack of reliable early-detection biomarkers. Circulating microRNAs (miRNAs) have emerged as promising non-invasive biomarkers for cancer detection and prognosis.

**Objective:**

This study aimed to computationally identify circulating miRNAs associated with early-stage OVCA using publicly available datasets and bioinformatics workflows.

**Methods:**

Differential expression analysis was performed on miRNA-Seq datasets from The Cancer Genome Atlas (TCGA) and Gene Expression Omnibus (GEO). Functional enrichment analysis and pathway annotation were performed using miEAA and PANTHER. A random forest-based machine-learning model was developed and optimized for miRNA biomarker classification.

**Results:**

Differential expression analysis revealed distinct miRNA signatures between OVCA and other cancer types (BRCA, CESC, UCEC, and COAD), as well as between OVCA and control samples. Stage-specific analysis identified key miRNAs, including *hsa-miR-29b-3p*, *hsa-miR-19b-3p*, and *hsa-miR-30e-5p*, consistently associated with early-stage OVCA. Functional enrichment analysis highlighted key pathways, including TP53 and VEGFA signaling, central to OVCA pathogenesis. The random forest classifier demonstrated robust performance with an accuracy of 91.67% and an area under the curve (AUC) of 0.991.

**Conclusion:**

This study identifies a panel of circulating miRNAs with significant diagnostic potential for early-stage OVCA. Integration of these miRNAs into clinical workflows could enhance early detection and improve patient outcomes. Further validation using independent cohorts is warranted.

## 1 Introduction

Ovarian cancer (OVCA) is the eighth most common cancer among women worldwide and remains the leading cause of gynecologic cancer-related deaths (Filho et al., 2024). Despite advancements in treatment protocols, late-stage diagnosis primarily contributes to the disease’s high mortality rate. More than 70% of OVCA cases are diagnosed at FIGO Stage III/IV, where the 5-year survival rate drops below 30% (World Ovarian Cancer Coalition, 2023). In contrast, early detection greatly improves outcomes, with survival rates over 90% when diagnosed at Stage I (Filho et al., 2024).

Current diagnostic tools, including CA-125 biomarker assays and transvaginal ultrasonography, lack the sensitivity and specificity required for reliable early-stage detection (Jacobs et al., 2016). Large-scale screening trials have demonstrated limited efficacy in reducing OVCA-related mortality, illustrating the urgent need to develop novel, accurate, and non-invasive diagnostic strategies.

MicroRNAs (miRNAs) have recently emerged as promising candidates for cancer biomarkers. These small, non-coding RNAs are key post-transcriptional regulators of gene expression and have been implicated in tumorigenesis, metastasis, and chemotherapy resistance (Hayes et al., 2014). Several studies have reported differential expression of miRNAs in OVCA, with specific miRNAs showing strong associations with disease stage and prognosis (Zhou et al., 2021; Huang et al., 2021). Their remarkable stability in bodily fluids, such as serum and plasma, makes them ideal candidates for non-invasive diagnostic assays. Despite growing evidence supporting their potential, the clinical application of miRNAs as diagnostic tools for early-stage OVCA remains limited, with insufficient validation across independent cohorts (Lin et al., 2022).

This study uses publicly available miRNA-Seq datasets and employs robust bioinformatics analyses to identify and validate circulating miRNAs associated with early-stage OVCA. Differential expression analysis, functional enrichment analysis, and machine-learning-based classification models were used to identify miRNAs with high diagnostic accuracy. These findings aim to contribute to the development of reliable noninvasive biomarkers for early-stage OVCA detection and lay the groundwork for their integration into clinical practice.

## 2 Materials and methods

### 2.1 Data collection and retrieval

Data for miRNA expression profiling were retrieved from two primary repositories: the Cancer Genome Atlas (TCGA) and the Gene Expression Omnibus (GEO). TCGA data were collected using filters for female gender, alive vital status, disease types including cystic, mucinous, and serous neoplasms, and primary sites limited to ovary, breast, corpus uteri, cervix uteri, and colon. The selected experimental strategy was miRNA-Seq, and metadata and miRNA expression quantification files were downloaded. GEO data were retrieved from the NCBI GEO database (https://www.ncbi.nlm.nih.gov/geo/) using the BioProject accession number PRJNA371423. This dataset included serum miRNA-Seq data from cancer, benign, borderline and control samples across different OVCA stages. We limited our study to serum samples annotated as serous ovarian carcinoma and non-cancer controls. Age, CA-125 levels, comorbidities and detailed treatment histories are not reported in the publicly available metadata for PRJNA371423, which limits the extent of clinical covariates that can be summarised for this cohort.

The final dataset comprised 325 samples from TCGA, including OVCA (n = 178), breast cancer (n = 14), cervix uteri cancer (n = 12), corpus uteri cancer (n = 98), and colon cancer (n = 23), and 37 samples from GEO, with 23 cancer samples and 14 control samples.

Stage-specific analyses were performed using the GEO serum cohort, whereas TCGA tumor samples were used for cancer-type comparisons and overall OVCA signatures without clinical stage stratification (Table 1).

**TABLE 1:** Summary of datasets, sample types, and sample distributions used across differential expression analyses.

Comparison	Data source	Sample type	Total samples (n)	Group distribution
OVCA vs. BRCA, CESC, UCEC, COAD	TCGA	Tumor tissue	325	178 OVCA, 147 other cancers
OVCA vs. Healthy control	GEO	Serum, serous	37	23 OVCA, 14 controls
Benign OVCA vs. Control	GEO	Serum, serous	32	18 benign, 14 controls
Borderline OVCA vs. Control	GEO	Serum, serous	31	17 borderline, 14 controls
Stage I/II OVCA vs. Control	GEO	Serum, serous	34	20 stage I/II OVCA, 14 controls
Stage III/IV OVCA vs. Control	GEO	Serum, serous	28	14 stage III/IV OVCA, 14 controls

### 2.2 Data preprocessing

Raw sequencing reads were preprocessed using a standardized pipeline. Quality control and adapter trimming were performed using Fastp (version 0.20.0). Genome mapping and annotation were carried out with mirPRo (version 1.1.4), aligning reads to the human reference genome (Homo_sapiens.GRCh38. dna.primary_assembly.fa.gz) and annotating known miRNAs using miRBase (version 22). Quantification of raw miRNA counts was performed using HTSeq (version 0.11.2) to ensure accurate downstream analysis.

### 2.3 Differential expression analysis

Differential expression analysis was conducted using the R Bioconductor package DESeq2. The dataset was normalized with the VSN package, and heatmaps were generated with the pheatmap package. Significantly differentially expressed miRNAs (DEMs) were identified based on an adjusted p-value threshold of less than 0.05 and a log2 fold change greater than 1, a conservative effect-size threshold commonly used in miRNA-Seq analyses. While smaller fold changes can be biologically significant, particularly in early-stage disease, they are also more susceptible to technical noise in miRNA-Seq data and batch effects across datasets. Volcano plots and heatmaps were employed to illustrate expression patterns.

### 2.4 Functional enrichment analysis

Functional enrichment analysis was conducted on differentially expressed miRNAs using the miRNA Enrichment Analysis and Annotation Tool (miEAA) and the PANTHER Overrepresentation Test. Gene Ontology (GO) annotations were used to examine biological processes associated with the identified miRNAs, and the PANTHER Pathway Database was used to examine pathway-level associations.

### 2.5 Prediction model construction

A random forest-based classifier was developed using the Waikato Environment for Knowledge Analysis (WEKA, version 3.8.6). Model evaluation was performed using 10-fold cross-validation, and optimization was achieved through the AutoWEKAClassifier tool. Performance metrics, including sensitivity, specificity, and area under the curve (AUC), were used to evaluate model accuracy and reliability. The original class distributions were retained and no over- or under-sampling was applied, as class imbalance was modest and model performance was evaluated using per-class metrics, including PRC area.

### 2.6 Statistical analysis

All statistical analyses were performed in R (version 4.1.0) using Bioconductor packages. False discovery rate (FDR) correction was applied using the Benjamini-Hochberg method to account for multiple comparisons and reduce false-positive findings.

## 3 Results

This study identified differentially expressed miRNAs (DEMs) in OVCA across cancer types, control samples, and disease stages. Key findings highlight miRNAs with diagnostic potential and their associated pathways. Extended differential expression, disease ontology, gene ontology, and pathway results are provided in the Supplementary Table.

### 3.1 Differential miRNA expression across cancer types

**FIGURE 1:**
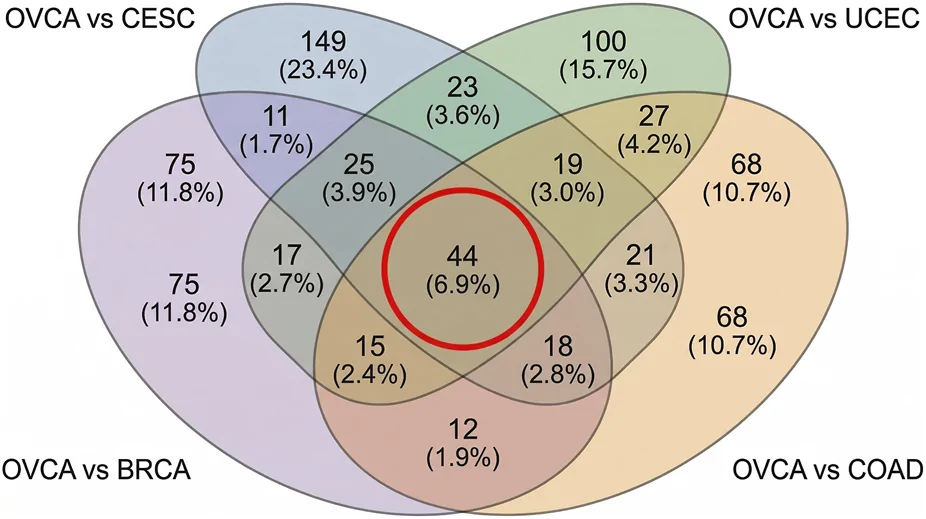
Venn diagram of common differentially expressed miRNAs across cancer types. This Venn diagram highlights miRNAs that are differentially expressed across OVCA, BRCA, CESC, UCEC, and COAD. Overlapping regions indicate shared miRNAs across these cancers. The number of shared miRNAs among them is 44, or 6.9%, as shown in the red circle.

Differential expression analysis revealed 276, 98, 429, and 427 DEMs in OVCA compared to breast cancer (BRCA), cervical squamous cell carcinoma (CESC), uterine corpus endometrial carcinoma (UCEC), and colon adenocarcinoma (COAD), respectively, using a Log2 fold change threshold greater than 1 and adjusted p-value of less than 0.05 (Table 2). The sample distributions across these cancer types are summarized in Table 3. The overlap of DEMs across all four cancer comparisons identified 44 shared miRNAs (6.9%), as illustrated in Figure 1.

**TABLE 2:** Summary of differentially expressed miRNAs (DEMs) identified across comparisons between OVCA and BRCA, CESC, UCEC, and COAD.

Differential analysis	Total DEMs	Upregulated DEMs	Downregulated DEMs
OVCA vs. BRCA	276	174	102
OVCA vs. CESC	98	62	36
OVCA vs. UCEC	429	260	169
OVCA vs. COAD	427	276	151

**TABLE 3:** Sample distribution across cancer type comparisons. OVCA samples include epithelial ovarian carcinomas of mixed histologies.

OVCA samples (n)	Matched comparison group
14	14 BRCA samples (breast cancer)
12	12 CESC samples (cervical squamous cell carcinoma)
98	98 UCEC samples (uterine corpus endometrial carcinoma)
23	23 COAD samples (colon adenocarcinoma)

### 3.2 Differential miRNA expression between OVCA and control samples

Comparison with control samples identified a set of miRNAs with significant dysregulation patterns, highlighting potential biomarkers for OVCA detection. (Table 4); the complete list of differentially expressed miRNAs is provided in Supplementary Table S2. Among the top ten candidates, *hsa-miR-320e* (Log2FC = 3.264; adj. p = 1.94 × 10^−6^) and *hsa-miR-10a-3p* (Log2FC = 2.618; adj. p = 3.43 × 10^−6^) demonstrated the most robust statistical significance, reinforcing their potential as diagnostic biomarkers. In contrast, *hsa-miR-3158-3p* was notably downregulated (Log2FC = −1.615), suggesting a potential tumor-suppressive role in OVCA.

**TABLE 4:** Top 10 differentially expressed miRNAs between OVCA and control samples (adjusted p-value < 0.05; Log2FC > 1).

#	miRNA	p-value	Adjusted p-value	Log2FC
1	*hsa-miR-320e*	9.69 × 10^−9^	1.94 × 10^−6^	+3.264
2	*hsa-miR-10a-3p*	2.05 × 10^−8^	3.43 × 10^−6^	+2.618
3	*hsa-miR-4488*	1.15 × 10^−7^	9.49 × 10^−6^	+3.129
4	*hsa-miR-200c-3p*	3.46 × 10^−5^	9.63 × 10^−4^	+2.555
5	*hsa-miR-320d-2*	1.01 × 10^−10^	1.02 × 10^−7^	+2.560
6	*hsa-miR-320d-1*	3.42 × 10^−9^	1.14 × 10^−6^	+2.182
7	*hsa-miR-1299*	4.19 × 10^−3^	3.93 × 10^−2^	+2.158
8	*hsa-miR-3158-3p*	6.68 × 10^−6^	2.48 × 10^−4^	−1.615
9	*hsa-miR-3182*	7.67 × 10^−4^	1.29 × 10^−2^	+2.139
10	*hsa-miR-200b-3p*	2.80 × 10^−3^	2.92 × 10^−2^	+1.851

### 3.3 Stage-specific miRNA expression in OVCA

**FIGURE 2:**
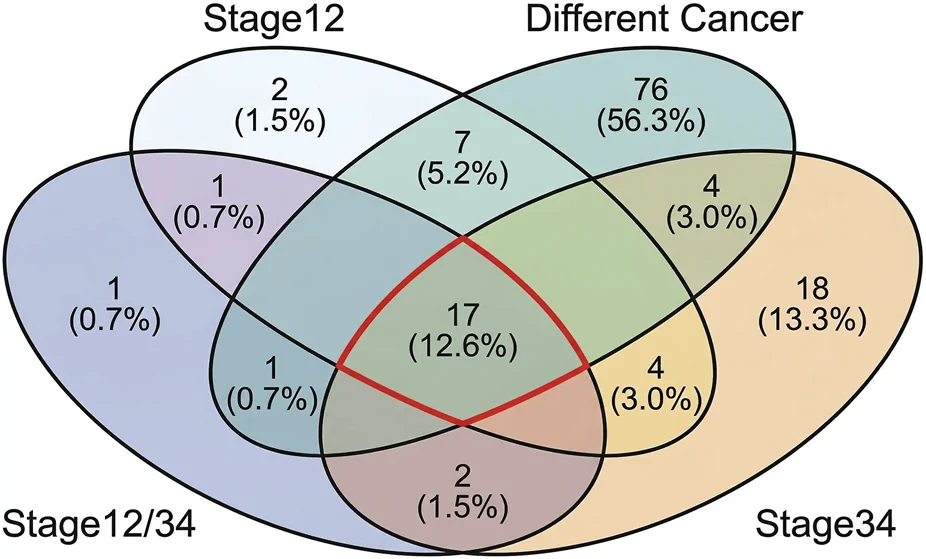
Venn diagram of exclusive miRNAs across OVCA comparisons. This Venn diagram highlights unique miRNAs associated with different OVCA comparisons, including BRCA, CESC, UCEC, and COAD, as well as stage-specific comparisons. We can see that in red, there are 17 or 12.6%, unique miRNAs to stage I/II OVCA.

Stage-specific analysis identified 19 miRNAs that are only associated with early-stage OVCA (Stage I/II). The top 10 most significant DEMs between Stage I/II OVCA and control samples, selected based on statistical significance (adjusted p-value < 0.05) and biological relevance (log2 fold change >1) are summarized in Table 5. The top 50 miRNAs are shown in Supplementary Table S5. The Venn diagram (Figure 2) highlights 17 unique miRNAs specific to early-stage OVCA. Key miRNAs including *hsa-miR-29b-3p*, *hsa-miR-19b-3*p, and *hsa-miR-30e-5p* were consistently identified as downregulated relative to controls, suggesting possible tumor-suppressive activity in early disease. The Venn diagram (Figure 2) highlights 17 miRNAs (12.6%) exclusive to early-stage OVCA comparisons. Tables 5, 6 present the 19 unique early-stage miRNAs and the top 10 DEMs between Stage I/II OVCA and controls, respectively.

**TABLE 5:** Unique microRNAs associated with early-Stage OVCA (Stage I/II). Statistics are based on comparison with control samples.

#	miRNA	p-value	Adjusted p-value	Log2FC
1	*hsa-miR-19b-1*	1.22e-05	1.21e-03	−1.210
2	*hsa-miR-19b-2*	1.25e-05	1.21e-03	−1.214
3	*hsa-miR-3909*	1.14e-04	4.51e-03	−0.890
4	*hsa-miR-19a-3p*	2.87e-04	7.53e-03	−0.876
5	*hsa-miR-450b-3p*	2.86e-04	7.53e-03	+2.073
6	*hsa-miR-19a*	2.87e-04	7.53e-03	−0.875
7	*hsa-mir-4433a*	2.01e-03	3.73e-02	+1.574
8	*hsa-miR-889*	2.00e-03	3.73e-02	+1.450
9	*hsa-miR-29b-3p*	1.83e-03	3.55e-02	−1.277
10	*hsa-mir-30e*	2.53e-03	4.43e-02	−0.610
11	*hsa-mir-335*	2.61e-03	4.50e-02	+0.838
12	*hsa-miR-93-3p*	7.28e-04	1.71e-02	−1.007
13	*hsa-miR-140-5p*	1.20e-03	2.61e-02	−0.589
14	*hsa-miR-30e-5p*	1.04e-03	2.31e-02	−0.773
15	*hsa-mir-4433a-3p*	2.79e-03	4.75e-02	+1.565
16	*hsa-miR-29b-1*	2.93e-03	4.80e-02	−1.211
17	*hsa-miR-29b-2*	2.94e-03	4.80e-02	−1.103
18	*hsa-mir-3909*	1.14e-04	4.51e-03	−0.890
19	*hsa-miR-19b-3p*	1.23e-05	1.21e-03	−1.217

**TABLE 6:** Top 10 differentially expressed miRNAs between Stage I/II OVCA and control samples (adjusted p-value < 0.05; Log2FC > 1).

#	miRNA	p-value	Adjusted p-value	Log2FC
1	*hsa-miR-10a-3p*	5.77 × 10^−7^	7.27 × 10^−4^	+2.618
2	*hsa-miR-320e*	1.92 × 10^−5^	1.21 × 10^−3^	+2.153
3	*hsa-miR-4488*	1.00 × 10^−5^	1.21 × 10^−3^	+1.979
4	*hsa-miR-200c*	3.79 × 10^−6^	1.21 × 10^−3^	+2.023
5	*hsa-miR-19b-1*	1.22 × 10^−5^	1.21 × 10^−3^	−1.210
6	*hsa-miR-19b-2*	1.25 × 10^−5^	1.21 × 10^−3^	−1.214
7	*hsa-miR-3158-1*	8.65 × 10^−6^	1.21 × 10^−3^	−1.380
8	*hsa-miR-3158-2*	8.65 × 10^−6^	1.21 × 10^−3^	−1.380
9	*hsa-miR-320d-2*	1.68 × 10^−5^	1.21 × 10^−3^	+1.129
10	*hsa-miR-532*	1.82 × 10^−5^	1.21 × 10^−3^	−1.007

To further investigate whether the identified biomarkers exhibited stage-specific expression patterns, a supplementary exploratory comparison was performed between Stage I/II and Stage III/IV OVCA samples. Several miRNAs were significantly differentially expressed between the two groups (Supplementary Table S9), including members of the miR-29 family and miR-30e. Notably, *hsa-miR-29b-3p* and *hsa-miR-30e-5p* showed significantly lower expression in Stage III/IV compared with Stage I/II disease, whereas *hsa-miR-19b-3p* did not differ significantly between stages. These findings suggest potential stage-associated expression changes for a subset of the identified biomarkers, although the analysis should be interpreted cautiously due to the limited cohort size.

### 3.4 Functional enrichment and pathway analysis

Functional enrichment analysis revealed that the identified miRNAs are implicated in key biological pathways central to OVCA pathogenesis, including TP53 signaling, VEGFA signaling, and TGF-beta signaling. Disease ontology analysis further mapped these miRNAs to specific OVCA subtypes, with each entry annotated by its disease ontology identifier, description, FDR value, and downstream target genes (Table 7); the complete disease ontology results are provided in Supplementary Table S7. Gene Ontology (GO) analysis identified cell proliferation, miRNA maturation, and epithelial cell proliferation as the most significantly enriched biological processes among downstream target genes, selected based on fold enrichment and statistical significance (Table 8); complete GO results are available in Supplementary Table S10. Together, these pathway associations underscore the mechanistic relevance of the identified miRNAs in OVCA pathogenesis and support their potential as both diagnostic and therapeutic targets.

**TABLE 7:** Key miRNAs and their disease ontology associations (FDR < 0.05), with downstream target genes.

DOID	miRNA	Description	FDR	Target genes
DOID:2151	*hsa-miR-19b-3p*	Malignant ovarian surface epithelial neoplasm	0.0207	PTEN, ESR1, BMPR2, TP53
DOID: 2152	*hsa-miR-29b-3p*	Ovary epithelial cancer	0.0013	VEGFA, ESR1, TGFB1, PTEN, MYC
DOID: 2394	*hsa-miR-30e-5p*	Ovarian cancer	0.0503	TP53
DOID: 3713	*hsa-miR-29b-3p*	Ovary adenocarcinoma	0.0051	VEGFA, PTEN
DOID: 4001	*hsa-miR-19b-3p*	Ovarian carcinoma	0.0207	PTEN, ESR1, BMPR2, TP53
DOID: 5683	*hsa-miR-29b-3p*	Hereditary breast/ovarian cancer	9.03E-05	VEGFA, ESR1, NCOA3, TGFB1, PTEN, MYC

**TABLE 8:** Top five Gene Ontology (GO) biological processes enriched in downstream target genes of differentially expressed miRNAs (FDR < 0.05).

GO term	Description	Fold enrichment	FDR
GO:0038091	Positive regulation of cell proliferation by VEGF-activated platelet-derived growth factor receptor signaling pathway	>100	4.46E-02
GO:1903800	Positive regulation of miRNA maturation	>100	1.25E-03
GO:0060391	Positive regulation of SMAD protein signal transduction	>100	2.24E-03
GO:2000637	Positive regulation of miRNA-mediated gene silencing	>100	2.99E-03
GO:0050679	Positive regulation of epithelial cell proliferation	49.26	1.64E-04

### 3.5 Machine learning prediction model performance

The RandomForest-based prediction model achieved an accuracy of 91.67%, an AUC of 0.991, and a sensitivity of 0.917. The RandomForest classification model was evaluated across three classes, Control, Early-stage OVCA, and Late-stage OVCA, using metrics including the True Positive Rate (TP Rate), False Positive Rate (FP Rate), Specificity, Precision, Recall, Receiver Operating Characteristic (ROC) Area, and Precision-Recall Curve (PRC) Area (Table 9). For the Early-stage OVCA class, the specificity was 0.964 and the precision was 0.947, with a PRC area of 0.982, indicating strong discriminative performance even in the presence of class imbalance. Notably, the Late-stage OVCA class achieved a perfect precision and specificity of 1.000, and the Control class a perfect Recall of 1.000, indicating high classification reliability across all groups. The weighted average performance across all classes included an accuracy of 91.67%, an AUC of 0.991, a specificity of 0.959, and a sensitivity of 0.917. Additional model parameters are detailed in Supplementary Table S12.

**TABLE 9:** Performance metrics of the random forest classification model across control, early-stage OVCA, and late-stage OVCA classes.

Class	TP rate	FP rate	Specificity	Precision	Recall	ROC area	PRC area
Control	1.000	0.088	0.912	0.824	1.000	0.991	0.974
Early-stage OVCA	0.900	0.036	0.964	0.947	0.900	0.985	0.982
Late-stage OVCA	0.857	0.000	1	1.000	0.857	0.999	0.995
Weighted Avg.	0.917	0.041	0.959	0.927	0.917	0.991	0.984

### 3.6 Integrated mechanistic model

**FIGURE 3:**
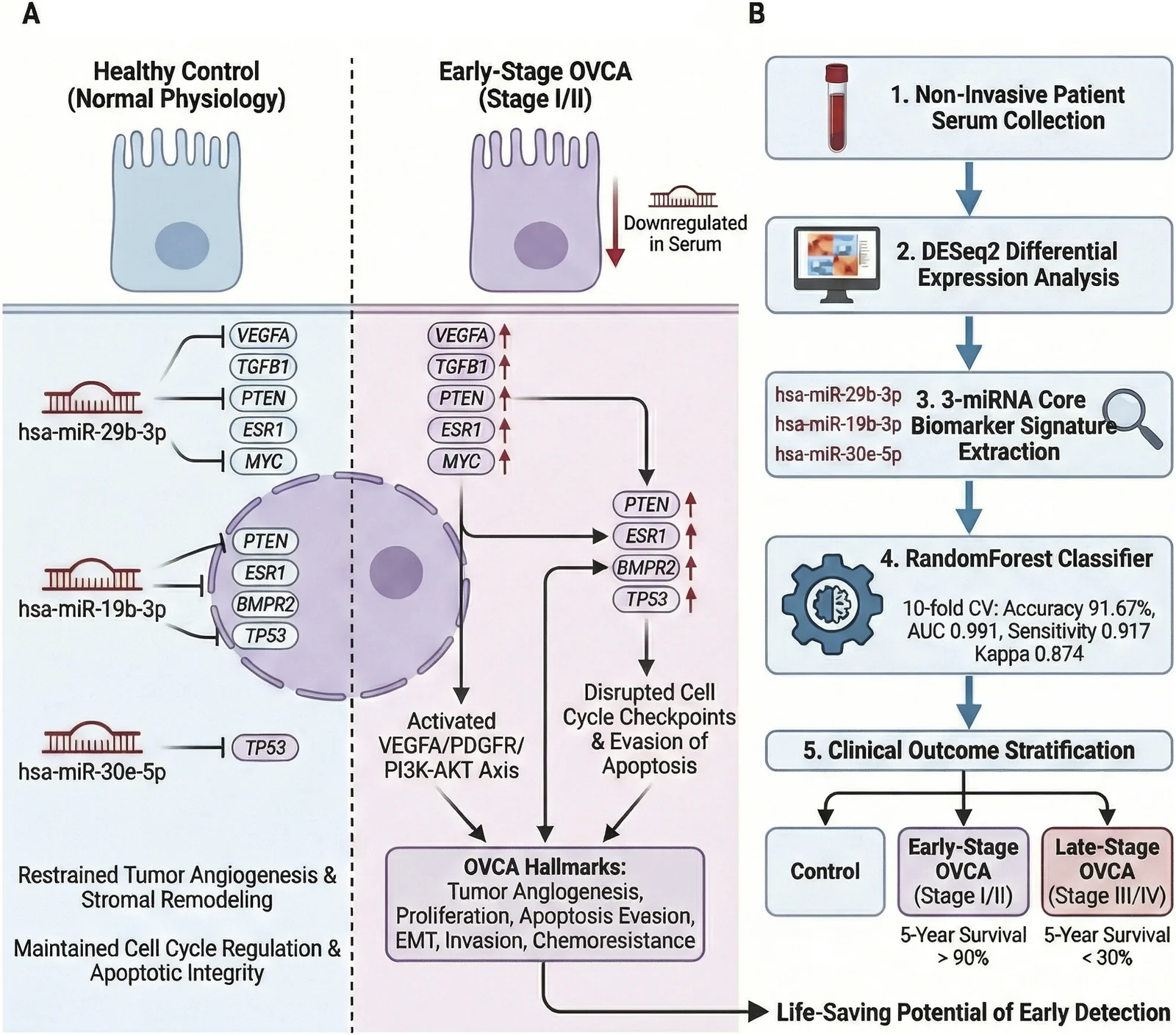
Mechanistic model depicting the roles of *hsa-miR-29b-3p*, *hsa-miR-19b-3p*, and *hsa-miR-30e-5p* in early-stage ovarian cancer development and non-invasive diagnosis. Figure generated using Nano Banana (Gemini image generation platform; Google DeepMind, 2023); see Acknowledgments for full AI disclosure. The authors confirm that the figure accurately reflects the data presented in this manuscript and is free from plagiarism. **(A)** Molecular mechanisms driving OVCA pathogenesis. **(B)** Translational diagnostic pipeline.

Based on the cumulative findings of the differential expression, pathway enrichment, and machine learning analyses, a mechanistic model was constructed to illustrate the roles of *hsa-miR-29b-3p*, *hsa-miR-19b-3p*, and *hsa-miR-30e-5p* in early-stage OVCA development and non-invasive diagnosis (Figure 3). All three miRNAs were identified as downregulated in patient serum at early-stage OVCA (Stage I/II) relative to healthy controls by DESeq2 differential expression analysis (Log_2_FC threshold >1; FDR-adjusted p-value < 0.05). Downregulation of *hsa-miR-29b-3p* derepresses VEGFA, TGFB1, PTEN, ESR1, and MYC, activating the VEGFA/PDGFR/PI3K-AKT axis and TGF-β/SMAD signaling cascade. Downregulation of *hsa-miR-19b-3p* derepresses PTEN, ESR1, BMPR2, and TP53, disrupting cell cycle regulation and apoptosis. Downregulation of *hsa-miR-30e-5p* further derepresses TP53, reinforcing cell cycle dysregulation. Together, these three pathways converge to promote tumor angiogenesis, epithelial cell proliferation, apoptosis evasion, epithelial-to-mesenchymal transition (EMT), peritoneal invasion, and chemotherapy resistance. The diagnostic pipeline illustrates the five-step clinical workflow: patient serum collection, DESeq2-based differential expression analysis, identification of the three-miRNA biomarker panel, RandomForest classification (WEKA v3.8.6; 10-fold cross-validation; accuracy = 91.67%; AUC = 0.991; sensitivity = 0.917; κ = 0.874), and clinical outcome stratification. Early detection at Stage I is associated with a 5-year survival rate exceeding 90%, compared with less than 30% at Stage III/IV.

## 4 Discussion

This study identified key differentially expressed miRNAs associated with OVCA across cancer types, control samples, and disease stages, emphasizing their diagnostic and functional significance. Through differential expression analysis, functional enrichment analysis, and machine-learning-based predictive modeling, *hsa-miR-29b-3p*, *hsa-miR-19b-3p*, and *hsa-miR-30e-5p* emerged as promising biomarkers for early-stage OVCA detection. Although these miRNAs have previously been linked to OVCA, the novelty of this work lies in integrating tumor and serum miRNA-Seq datasets, incorporating cross-cancer comparisons during biomarker selection, and explicitly focusing on earlier disease stages.

### 4.1 Differential miRNA expression across cancer types

Differential expression analysis revealed distinct miRNA expression patterns between OVCA and other cancer types (Table 3). Although 44 miRNAs (6.9%) were shared across comparisons, suggesting involvement in common oncogenic processes, most differentially expressed miRNAs were unique to OVCA, highlighting OVCA-specific regulatory mechanisms. Prior studies have demonstrated that *hsa-miR-29b-3p* and *hsa-miR-19b-3p* play key roles in apoptosis regulation, immune response, and angiogenesis across multiple cancer types (Wang et al., 2022; Zhou et al., 2021). These OVCA-specific expression patterns nonetheless suggest distinct molecular processes that warrant further investigation.

### 4.2 Differential miRNA expression between OVCA and control samples

Comparison between OVCA and control samples highlighted a subset of miRNAs with significant dysregulation (Table 4). Among the top candidates, *hsa-miR-320e* (Log2FC = 3.264, adj. p = 1.94 × 10^−8^) and *hsa-miR-10a-3p* (Log2FC = 2.618; adj. p = 3.43 × 10^−6^) demonstrated some of the strongest expression differences, reinforcing their potential as diagnostic biomarkers. In addition, *hsa-miR-4488* and *hsa-miR-200c-3p* displayed notable dysregulation, with log2 fold changes of 3.129 and 2.555, respectively. These miRNAs are stable in serum and have been linked to cancer-relevant pathways (Luo et al., 2022), underscoring their potential as non-invasive diagnostic biomarkers for OVCA.

### 4.3 Stage-specific miRNA expression patterns

Stage-specific analysis revealed 19 miRNAs uniquely associated with early-stage OVCA (Stage I/II) (Tables 5, 6; Figure 2). Among these, *hsa-miR-29b-3p*, *hsa-miR-19b-3p*, and *hsa-miR-30e-5p* were consistently linked to early-stage disease, aligning with their known roles in immune regulation, apoptosis, and tumor progression (Lin et al., 2022; Xu et al., 2020). Notably, *hsa-miR-29b-3p* targets the VEGFA signaling pathway, while *hsa-miR-19b-3p* disrupts tumor suppressor signaling through interactions with PTEN, ESR1, BMPR2 and TP53 (Zhang et al., 2021). These characteristics motivated the selection of *hsa-miR-29b-3p*, *hsa-miR-19b-3p*, and *hsa-miR-30e-5p* as the three-miRNA panel for early-stage OVCA detection.

### 4.4 Functional enrichment and pathway analysis

Functional enrichment analysis highlighted the involvement of key biological pathways, including TP53 signaling, VEGFA, and TGF-beta signaling (Tables 7, 8), all of which are known contributors to ovarian tumorigenesis. The TP53 pathway, modulated by *hsa-miR-19b-3p* and *hsa-miR-29b-3p*, plays a pivotal role in tumor suppression, cell cycle regulation, and apoptosis (Dai et al., 2021). Dysregulation of the VEGFA pathway, targeted by *hsa-miR-29b-3p*, promotes angiogenesis and contributes to tumor growth and metastasis (Luo et al., 2022). These findings align with existing literature supporting the biological relevance of these pathways and their potential as therapeutic targets in OVCA management. Based on these findings, we propose a mechanistic model depicting the roles of the three key miRNAs in early-stage OVCA pathogenesis (Figure 3).

### 4.5 Machine learning model and diagnostic performance

The RandomForest-based machine learning model demonstrated strong predictive power, achieving an accuracy of 91.67%, an AUC of 0.991, a specificity of 0.959, and a sensitivity of 0.917 (Table 9). These metrics indicate that the miRNA panel *hsa-miR-29b-3p*, *hsa-miR-19b-3p*, and *hsa-miR-30e-5p* serves as a dependable classifier for early-stage OVCA detection. Prior research employing machine learning models for miRNA-based OVCA classification has reported comparable performance (Shen et al., 2022; Gao et al., 2022). However, external validation using independent datasets is necessary to confirm these observations and ensure reproducibility across diverse patient populations.

### 4.6 Clinical and diagnostic implications

The miRNAs identified in this study, particularly *hsa-miR-29b-3p*, *hsa-miR-19b-3p*, and *hsa-miR-30e-5p*, hold significant promise as non-invasive serum biomarkers for early detection of OVCA. That being said, CA-125 remains the most widely used serum biomarker for OVCA. Although published studies have generally reported lower diagnostic performance for CA-125 in early-stage OVCA than observed for miRNA panels (Jacobs et al., 2016), any comparison is necessarily indirect because CA-125 measurements were not available for the PRJNA371423 cohort. Consequently, these differences should be interpreted with caution, and a formal comparison will require prospective cohorts with paired CA-125 and miRNA measurements. Their integration into clinical workflows could enhance diagnostic accuracy, reduce reliance on invasive biopsy procedures, and ultimately improve patient outcomes. In addition to their diagnostic value, the enrichment of pathways such as TP53 and VEGFA signaling suggests their potential utility as therapeutic targets in combination with existing OVCA treatment strategies.

### 4.7 Study limitations and future directions

Despite all the insights gained, several limitations must be acknowledged. The reliance on publicly available datasets may introduce selection and batch-effect biases, and external validation in independent clinical cohorts is essential to confirm the findings. The sample size, particularly for early-stage OVCA, remains relatively small, which may limit the statistical power of stage-specific analysis. Biological differences between tissue and serum also need to be considered when comparing results across cohorts. Although our filtering and normalization strategies reduce technical batch effects, they cannot fully eliminate these differences. Additionally, limited clinical metadata, including CA-125 levels and comorbidity information, restricted comprehensive clinical characterization of the cohorts, while the predominance of serous epithelial tumors may limit the generalizability of these findings to other OVCA histological subtypes. Because the primary analyses focused on comparisons between OVCA and non-cancer controls, the ability of the proposed miRNA panel to distinguish OVCA from benign ovarian masses was not fully evaluated. Furthermore, experimental validation of the identified miRNAs and their functional roles in OVCA pathogenesis is needed to bridge the gap between bioinformatics predictions and clinical application.

Future research should focus on: (1) validating these miRNAs in prospective, diverse patient cohorts; (2) developing standardized, high-throughput diagnostic assays, incorporating this miRNA panel; and exploring their therapeutic potential in preclinical and clinical settings.

## 5 Conclusion

This study identified *hsa-miR-29b-3p*, *hsa-miR-19b-3p*, and *hsa-miR-30e-5p* as key differentially expressed miRNAs across OVCA, control samples, and disease stages. Additionally, *hsa-miR-320e*, *hsa-miR-10a-3p*, *hsa-miR-4488*, and *hsa-miR-200c-3p* were identified as highly promising biomarkers in comparisons between OVCA and control samples, with significant involvement in pathways related to apoptosis, angiogenesis, and tumor suppression. Functional enrichment analysis highlighted critical associations with the TP53 and VEGFA pathways, emphasizing their biological and diagnostic significance. The RandomForest-based machine-learning model further demonstrated robust diagnostic performance (accuracy = 91.67%; AUC = 0.991), underscoring the potential of this miRNA panel as a non-invasive diagnostic tool for early-stage OVCA.

## Data Availability

The data analyzed in this study were obtained from publicly accessible repositories. TCGA miRNA-Seq data are available via the GDC Data Portal (https://portal.gdc.cancer.gov/). GEO data were retrieved under BioProject accession PRJNA371423 (https://www.ncbi.nlm.nih.gov/bioproject/PRJNA371423). Supplementary data tables are included within the article and its Supplementary Material. The original analysis scripts used in this study are available from the corresponding authors upon reasonable request.
