# Supplementary material for: A circulating three-miRNA panel (hsa-miR-29b-3p, hsa-miR-19b-3p, hsa-miR-30e-5p) for early-stage ovarian cancer detection: a machine-learning bioinformatics approach
Source: Front Genet. 2026 Aug 12;17:1827636. doi: 10.3389/fgene.2026.1827636 (PMC13506143; doi:10.3389/fgene.2026.1827636)
Supplement: Supplementary file 1 [file Table1.docx]

# Supplementary Data

## Supplementary Section S1: Extended Differential Expression Analysis

### Supplementary Table S1. Common miRNAs Across Comparisons. The table lists common miRNAs identified across multiple pairwise comparisons of cancer types. The comparisons include OVCA vs. BRCA (Res2), OVCA vs. CESC (Res3), UCEC (Res4), and OVCA vs. COAD (Res5). Columns show log fold change (LFC) and adjusted p-values (padj) for each comparison.

| **miRNA** | **res2_LFC** | **res2_padj** | **res3_LFC** | **res3_padj** | **res4_LFC** | **res4_padj** | **res5_LFC** | **res5_padj** |
| --- | --- | --- | --- | --- | --- | --- | --- | --- |
| ***hsa-mir-1-1*** | -3.977 | 3.674E-04 | -4.018 | 3.335E-03 | -4.325 | 4.452E-20 | -3.969 | 5.21E-05 |
| ***hsa-mir-1-2*** | -4.112 | 7.237E-04 | -4.067 | 3.335E-03 | -4.760 | 2.659E-24 | -3.752 | 1.499E-04 |
| ***hsa-mir-1229*** | 1.706 | 1.084E-02 | 2.054 | 3.952E-02 | 1.319 | 2.090E-06 | 2.702 | 1.859E-05 |
| ***hsa-mir-1237*** | 2.874 | 8.399E-03 | 2.854 | 2.063E-02 | 1.549 | 3.948E-06 | 2.267 | 9.325E-03 |
| ***hsa-mir-1254-1*** | 1.413 | 1.208E-02 | 1.819 | 3.952E-02 | 1.622 | 4.082E-12 | 2.267 | 3.076E-04 |
| ***hsa-mir-1254-2*** | 1.366 | 2.074E-02 | 1.941 | 2.172E-02 | 1.733 | 4.912E-13 | 2.341 | 1.102E-04 |
| ***hsa-mir-1270*** | 2.954 | 2.656E-04 | 2.658 | 2.546E-02 | 1.897 | 1.163E-09 | 3.753 | 7.54E-07 |
| ***hsa-mir-129-1*** | 2.778 | 7.503E-03 | 2.596 | 1.484E-02 | 1.575 | 3.632E-08 | 2.971 | 3.16E-05 |
| ***hsa-mir-129-2*** | 2.725 | 3.119E-03 | 2.538 | 5.135E-03 | 1.568 | 3.179E-08 | 2.886 | 1.86E-05 |
| ***hsa-mir-153-2*** | -5.146 | 5.138E-03 | -4.319 | 1.747E-02 | -2.071 | 8.516E-06 | -2.779 | 1.968E-02 |
| ***hsa-mir-196a-1*** | -3.694 | 2.656E-04 | -4.478 | 1.521E-04 | -1.945 | 3.412E-06 | -2.781 | 5.366E-04 |
| ***hsa-mir-196a-2*** | -3.753 | 3.257E-04 | -4.752 | 1.521E-04 | -1.970 | 5.780E-06 | -2.841 | 4.807E-04 |
| ***hsa-mir-199a-1*** | -1.832 | 3.925E-03 | -1.747 | 4.485E-02 | -1.504 | 1.47E-11 | -2.141 | 1.19E-06 |
| ***hsa-mir-199a-2*** | -1.909 | 4.544E-03 | -1.790 | 4.801E-02 | -1.599 | 7.76E-12 | -2.410 | 3.43E-07 |
| ***hsa-mir-2355*** | -2.091 | 6.979E-03 | -2.078 | 1.949E-02 | -1.235 | 4.00E-06 | -1.810 | 6.172E-04 |
| ***hsa-mir-30b*** | -1.649 | 1.087E-02 | -1.808 | 2.846E-02 | -1.577 | 8.52E-12 | -1.223 | 1.042E-03 |
| ***hsa-mir-3615*** | 1.575 | 1.647E-03 | 1.795 | 3.920E-03 | 1.341 | 6.28E-13 | 2.151 | 3.431E-07 |
| ***hsa-mir-3622a*** | 3.907 | 2.656E-04 | 3.170 | 2.172E-02 | 1.360 | 1.985E-04 | 3.659 | 6.621E-04 |
| ***hsa-mir-369*** | -1.226 | 1.993E-02 | -1.759 | 8.109E-03 | -1.037 | 1.706E-04 | -1.505 | 1.378E-03 |
| ***hsa-mir-378a*** | 2.745 | 9.043E-06 | 1.307 | 2.788E-02 | 1.331 | 2.01E-08 | 1.450 | 6.39E-05 |
| ***hsa-mir-3928*** | 2.091 | 1.606E-05 | 2.386 | 5.491E-03 | 1.228 | 1.25E-08 | 2.262 | 1.92E-06 |
| ***hsa-mir-424*** | -1.716 | 5.183E-03 | -3.224 | 2.15E-06 | -1.965 | 2.08E-15 | -2.114 | 1.02E-05 |
| ***hsa-mir-4443*** | 1.676 | 1.995E-02 | 2.922 | 1.003E-02 | 1.172 | 1.34E-05 | 1.683 | 3.690E-03 |
| ***hsa-mir-450a-2*** | -1.997 | 2.254E-02 | -3.236 | 3.335E-03 | -2.192 | 4.32E-10 | -1.532 | 4.120E-02 |
| ***hsa-mir-4523*** | 3.862 | 5.086E-04 | 2.847 | 1.741E-02 | 1.197 | 2.27E-05 | 2.642 | 5.422E-04 |
| ***hsa-mir-483*** | 2.921 | 1.193E-02 | 3.415 | 3.335E-03 | 1.124 | 8.828E-03 | 2.026 | 3.373E-02 |
| ***hsa-mir-5091*** | 2.260 | 2.304E-03 | 2.078 | 3.133E-02 | 1.604 | 4.44E-09 | 1.562 | 4.837E-03 |
| ***hsa-mir-542*** | -3.218 | 7.60E-05 | -2.801 | 2.063E-02 | -2.426 | 9.86E-14 | -1.973 | 1.441E-03 |
| ***hsa-mir-582*** | -3.392 | 1.16E-05 | -2.947 | 2.339E-02 | -1.159 | 5.065E-04 | -1.947 | 1.118E-02 |
| ***hsa-mir-588*** | 3.527 | 7.102E-04 | 3.622 | 3.335E-03 | 1.713 | 3.14E-05 | 3.657 | 5.45E-06 |
| ***hsa-mir-6509*** | 3.135 | 5.72E-05 | 2.335 | 2.173E-03 | 1.095 | 1.36E-07 | 2.840 | 7.54E-07 |
| ***hsa-mir-6514*** | 1.584 | 2.808E-03 | 1.563 | 3.782E-02 | 1.071 | 5.04E-08 | 1.852 | 1.689E-04 |
| ***hsa-mir-653*** | -2.627 | 2.030E-02 | -2.300 | 3.250E-02 | -2.582 | 7.35E-11 | -2.411 | 2.729E-03 |
| ***hsa-mir-671*** | 1.131 | 1.042E-03 | 1.333 | 6.650E-03 | 1.079 | 3.36E-11 | 1.890 | 4.86E-08 |
| ***hsa-mir-6746*** | 3.081 | 1.857E-03 | 3.320 | 2.788E-02 | 1.373 | 1.706E-04 | 2.778 | 1.634E-03 |
| ***hsa-mir-6747*** | 2.564 | 3.257E-04 | 2.162 | 1.741E-02 | 1.727 | 4.348E-13 | 2.798 | 2.03E-06 |
| ***hsa-mir-6794*** | 3.387 | 2.704E-03 | 4.083 | 1.741E-02 | 2.368 | 6.995E-09 | 3.141 | 1.413E-03 |
| ***hsa-mir-6802*** | 2.506 | 1.51E-05 | 1.927 | 2.639E-02 | 1.361 | 2.024E-09 | 2.568 | 1.19E-06 |
| ***hsa-mir-6842*** | 2.373 | 4.97E-05 | 2.476 | 3.845E-04 | 1.269 | 9.570E-09 | 1.666 | 6.99E-05 |
| ***hsa-mir-6843*** | 3.838 | 6.230E-04 | 4.208 | 3.335E-03 | 1.580 | 3.383E-05 | 3.885 | 4.50E-05 |
| ***hsa-mir-6875*** | 3.151 | 1.686E-04 | 2.883 | 3.335E-03 | 1.690 | 4.296E-10 | 3.469 | 3.52E-08 |
| ***hsa-mir-6892*** | 2.639 | 2.470E-04 | 3.149 | 2.173E-03 | 1.366 | 2.083E-08 | 2.740 | 6.75E-07 |
| ***hsa-mir-885*** | 4.124 | 2.091E-04 | 3.481 | 3.335E-03 | 1.463 | 3.150E-07 | 5.762 | 2.60E-09 |
| ***hsa-mir-92b*** | 2.534 | 2.091E-04 | 1.633 | 8.087E-03 | 1.485 | 6.938E-12 | 2.834 | 1.78E-06 |

**Supplementary Table S2. Top 50 Differentially Expressed miRNAs Between OVCA and Control.** Top 50 miRNAs differentially expressed between OVCA and control samples, selected based on statistical significance (adjusted p-value < 0.05) and biological relevance (log2 fold change > 1).

|  | **miRNA** | **p-value** | **Adjusted p-value** | **LFC** |
| --- | --- | --- | --- | --- |
| **1** | *hsa-miR-200b-3p* | 2.7979e-03 | 2.9203e-02 | 1.8507 |
| **2** | *hsa-mir-200b* | 2.1042e-04 | 6.2734e-03 | 1.4628 |
| **3** | *hsa-miR-10a-3p* | 2.0545e-08 | 3.4310e-06 | 2.6177 |
| **4** | *hsa-miR-200c-3p* | 3.4605e-05 | 9.6316e-04 | 2.5550 |
| **5** | *hsa-miR-3158-3p* | 6.6843e-06 | 2.4816e-04 | -1.6151 |
| **6** | *hsa-mir-200c* | 3.7943e-06 | 1.2098e-03 | 2.0229 |
| **7** | *hsa-mir-3158-1* | 6.9279e-06 | 2.4816e-04 | -1.6128 |
| **8** | *hsa-mir-3158-2* | 6.9279e-06 | 2.4816e-04 | -1.6128 |
| **9** | *hsa-miR-320e* | 1.9203e-05 | 1.2098e-03 | 2.1526 |
| **10** | *hsa-mir-320e* | 1.9203e-05 | 1.2098e-03 | 2.1526 |
| **11** | *hsa-miR-12136* | 2.7078e-06 | 1.4596e-04 | 4.0279 |
| **12** | *hsa-miR-4488* | 1.1547e-07 | 9.4910e-06 | 3.1288 |
| **13** | *hsa-miR-532-5p* | 3.3503e-06 | 1.4596e-04 | -1.3070 |
| **14** | *hsa-mir-12136* | 2.7078e-06 | 1.4596e-04 | 4.0279 |
| **15** | *hsa-mir-4488* | 1.0016e-05 | 1.2098e-03 | 1.9790 |
| **16** | *hsa-mir-532* | 1.8218e-05 | 1.2098e-03 | -1.0067 |
| **17** | *hsa-miR-625-3p* | 1.4236e-05 | 1.2098e-03 | 1.0319 |
| **18** | *hsa-mir-625* | 1.4236e-05 | 1.2098e-03 | 1.0319 |
| **19** | *hsa-miR-6131* | 1.8842e-05 | 1.2098e-03 | 1.3656 |
| **20** | *hsa-mir-320d-2* | 1.0142e-10 | 1.0163e-07 | 2.5603 |
| **21** | *hsa-mir-6131* | 1.8842e-05 | 1.2098e-03 | 1.3656 |
| **22** | *hsa-miR-26a-5p* | 4.1336e-04 | 1.0284e-02 | 0.7017 |
| **23** | *hsa-mir-26a-1* | 4.1624e-04 | 1.0284e-02 | 0.7010 |
| **24** | *hsa-mir-26a-2* | 4.1166e-04 | 1.0284e-02 | 0.7023 |
| **25** | *hsa-miR-7706* | 4.5088e-04 | 8.5218e-03 | -1.2377 |
| **26** | *hsa-miR-3909* | 1.1447e-04 | 4.5073e-03 | -0.8899 |
| **27** | *hsa-mir-3909* | 1.1447e-04 | 4.5073e-03 | -0.8899 |
| **28** | *hsa-miR-941* | 1.2815e-03 | 1.5844e-02 | -0.7156 |
| **29** | *hsa-mir-941-1* | 1.2815e-03 | 1.5844e-02 | -0.7098 |
| **30** | *hsa-mir-941-2* | 1.2815e-03 | 1.5844e-02 | -0.7098 |
| **31** | *hsa-mir-941-3* | 1.2815e-03 | 1.5844e-02 | -0.7098 |
| **32** | *hsa-mir-941-4* | 1.2815e-03 | 1.5844e-02 | -0.7098 |
| **33** | *hsa-mir-941-5* | 1.2815e-03 | 1.5844e-02 | -0.7098 |
| **34** | *hsa-miR-106b-3p* | 1.0883e-04 | 4.5073e-03 | -0.6744 |
| **35** | *hsa-mir-106b* | 1.0883e-04 | 4.5073e-03 | -0.6744 |
| **36** | *hsa-mir-320c-2* | 1.7280e-07 | 1.2367e-05 | 1.9911 |
| **37** | *hsa-miR-140-3p* | 5.3393e-05 | 2.5875e-03 | -0.8661 |
| **38** | *hsa-miR-320c* | 5.7759e-05 | 2.6954e-03 | 0.9242 |
| **39** | *hsa-mir-140* | 5.3393e-05 | 2.5875e-03 | -0.8661 |
| **40** | *hsa-miR-320d* | 4.0572e-05 | 2.5875e-03 | 2.1821 |
| **41** | *hsa-miR-4470* | 2.8579e-04 | 7.5318e-03 | 2.0722 |
| **42** | *hsa-mir-4470* | 2.8579e-04 | 7.5318e-03 | 2.0722 |
| **43** | *hsa-mir-320c-1* | 9.3364e-08 | 9.3551e-06 | 2.0408 |
| **44** | *hsa-miR-1255b-5p* | 2.7732e-05 | 8.6836e-04 | -1.8406 |
| **45** | *hsa-miR-25-3p* | 6.9346e-06 | 2.4816e-04 | -1.5613 |
| **46** | *hsa-mir-25* | 2.9735e-03 | 4.8033e-02 | -0.8689 |
| **47** | *hsa-mir-1255b-1* | 3.1957e-05 | 9.4178e-04 | -1.8132 |
| **48** | *hsa-mir-1255b-2* | 3.0144e-05 | 9.1528e-04 | -1.8197 |
| **49** | *hsa-mir-320d-1* | 3.4205e-09 | 1.1424e-06 | 2.1821 |
| **50** | *hsa-mir-7706* | 4.5088e-04 | 8.5218e-03 | -1.2377 |

**Supplementary Table S3. Top 50 Differentially Expressed miRNAs Between Benign OVCA and Control.** Top 50 miRNAs differentially expressed between Benign OVCA and control samples, selected based on statistical significance (adjusted p-value < 0.05) and biological relevance (log2 fold change > 1).

|  | **miRNA** | **p-value** | **Adjusted p-value** | **LFC** |
| --- | --- | --- | --- | --- |
| **1** | *hsa-mir-12136* | 5.2571e-07 | 7.6228e-05 | 4.0077 |
| **2** | *hsa-mir-3158-1* | 3.9684e-07 | 7.6228e-05 | -1.7023 |
| **3** | *hsa-mir-3158-2* | 3.9684e-07 | 7.6228e-05 | -1.7023 |
| **4** | *hsa-mir-532* | 4.1158e-07 | 7.6228e-05 | -1.1432 |
| **5** | *hsa-miR-12136* | 5.2571e-07 | 7.6228e-05 | 4.0077 |
| **6** | *hsa-miR-3158-3p* | 3.5454e-07 | 7.6228e-05 | -1.7039 |
| **7** | *hsa-miR-532-5p* | 4.1158e-07 | 7.6228e-05 | -1.1432 |
| **8** | *hsa-mir-6131* | 5.9470e-06 | 6.7069e-04 | 1.4496 |
| **9** | *hsa-miR-6131* | 5.9470e-06 | 6.7069e-04 | 1.4496 |
| **10** | *hsa-miR-10a-3p* | 1.5419e-05 | 1.5651e-03 | 1.6659 |
| **11** | *hsa-let-7b* | 3.0684e-05 | 2.2246e-03 | -1.1309 |
| **12** | *hsa-mir-206* | 2.7125e-05 | 2.2246e-03 | -1.8062 |
| **13** | *hsa-let-7b-5p* | 3.0684e-05 | 2.2246e-03 | -1.1309 |
| **14** | *hsa-miR-206* | 2.7125e-05 | 2.2246e-03 | -1.8062 |
| **15** | *hsa-mir-25* | 4.2650e-05 | 2.7056e-03 | -1.3469 |
| **16** | *hsa-miR-25-3p* | 4.2650e-05 | 2.7056e-03 | -1.3469 |
| **17** | *hsa-miR-1255b-5p* | 4.5971e-05 | 2.7448e-03 | -1.5948 |
| **18** | *hsa-mir-1255b-1* | 5.4677e-05 | 2.8133e-03 | -1.5787 |
| **19** | *hsa-mir-1255b-2* | 5.4677e-05 | 2.8133e-03 | -1.5787 |
| **20** | *hsa-mir-1273h* | 5.5434e-05 | 2.8133e-03 | 0.8929 |
| **21** | *hsa-miR-1273h-3p* | 5.8906e-05 | 2.8471e-03 | 0.9038 |
| **22** | *hsa-mir-326* | 7.6341e-05 | 3.2286e-03 | 1.1405 |
| **23** | *hsa-miR-199a-5p* | 7.3838e-05 | 3.2286e-03 | 1.0190 |
| **24** | *hsa-miR-326* | 7.6341e-05 | 3.2286e-03 | 1.1405 |
| **25** | *hsa-mir-26a-1* | 1.2853e-04 | 4.8318e-03 | 0.7314 |
| **26** | *hsa-mir-26a-2* | 1.2371e-04 | 4.8318e-03 | 0.7339 |
| **27** | *hsa-miR-26a-5p* | 1.2590e-04 | 4.8318e-03 | 0.7326 |
| **28** | *hsa-mir-7976* | 1.4042e-04 | 4.9146e-03 | -1.1424 |
| **29** | *hsa-miR-7976* | 1.4042e-04 | 4.9146e-03 | -1.1424 |
| **30** | *hsa-mir-625* | 1.5103e-04 | 4.9451e-03 | 0.8245 |
| **31** | *hsa-miR-625-3p* | 1.5103e-04 | 4.9451e-03 | 0.8245 |
| **32** | *hsa-miR-151a-5p* | 2.2281e-04 | 7.0672e-03 | 1.0128 |
| **33** | *hsa-mir-1224* | 2.7540e-04 | 7.8739e-03 | -1.4448 |
| **34** | *hsa-mir-127* | 2.8750e-04 | 7.8739e-03 | 1.9979 |
| **35** | *hsa-mir-493* | 2.9479e-04 | 7.8739e-03 | 1.3149 |
| **36** | *hsa-miR-1224-5p* | 2.7540e-04 | 7.8739e-03 | -1.4448 |
| **37** | *hsa-miR-127-3p* | 2.8750e-04 | 7.8739e-03 | 1.9979 |
| **38** | *hsa-miR-493-5p* | 2.9480e-04 | 7.8739e-03 | 1.4121 |
| **39** | *hsa-mir-4504* | 3.3816e-04 | 8.5809e-03 | -1.7598 |
| **40** | *hsa-miR-4504* | 3.3816e-04 | 8.5809e-03 | -1.7598 |
| **41** | *hsa-miR-1307-5p* | 4.8903e-04 | 1.2106e-02 | 0.6778 |
| **42** | *hsa-mir-11400* | 6.3270e-04 | 1.4935e-02 | 1.0001 |
| **43** | *hsa-miR-11400* | 6.3270e-04 | 1.4935e-02 | 1.0001 |
| **44** | *hsa-mir-199a-1* | 6.9458e-04 | 1.5308e-02 | 0.8558 |
| **45** | *hsa-mir-199a-2* | 6.9458e-04 | 1.5308e-02 | 0.8558 |
| **46** | *hsa-mir-23b* | 7.3901e-04 | 1.5308e-02 | 0.9655 |
| **47** | *hsa-mir-6852* | 7.0489e-04 | 1.5308e-02 | 0.9229 |
| **48** | *hsa-miR-25-5p* | 7.3279e-04 | 1.5308e-02 | -0.9114 |
| **49** | *hsa-miR-6852-5p* | 7.0489e-04 | 1.5308e-02 | 0.9229 |
| **50** | *hsa-mir-487b* | 1.1264e-03 | 2.0097e-02 | 2.2052 |

**Supplementary Table S4. Top Differentially Expressed miRNAs Between Borderline OVCA and Control.**

|  | **miRNAs** | **p-value** | **Adjusted p-value** | **LFC** |
| --- | --- | --- | --- | --- |
| **1** | *hsa-mir-12136* | 1.8729e-05 | 1.71e-02 | 3.6132 |
| **2** | *hsa-mir-625* | 2.7998e-05 | 1.71e-02 | 0.9927 |
| **3** | *hsa-miR-12136* | 1.8729e-05 | 1.71e-02 | 3.6132 |
| **4** | *hsa-miR-625-3p* | 2.7998e-05 | 1.71e-02 | 0.9927 |

**Supplementary Table S5. Top 50 Differentially Expressed miRNAs Between Stage I/II OVCA and Control.** Top 50 miRNAs differentially expressed between Stage I/II OVCA and control samples, selected based on statistical significance (adjusted p-value < 0.05) and biological relevance (log2 fold change > 1).

|  | **miRNA** | **p-value** | **padj** | **log2FoldChange** |
| --- | --- | --- | --- | --- |
| **1** | *hsa-miR-10a-3p* | 5.7730e-07 | 7.2740e-04 | 2.2363 |
| **2** | *hsa-mir-19b-1* | 1.2210e-05 | 1.2098e-03 | -1.2101 |
| **3** | *hsa-mir-19b-2* | 1.2538e-05 | 1.2098e-03 | -1.2143 |
| **4** | *hsa-mir-200c* | 3.7943e-06 | 1.2098e-03 | 2.0229 |
| **5** | *hsa-mir-3158-1* | 8.6528e-06 | 1.2098e-03 | -1.3799 |
| **6** | *hsa-mir-3158-2* | 8.6528e-06 | 1.2098e-03 | -1.3799 |
| **7** | *hsa-mir-320d-2* | 1.6814e-05 | 1.2098e-03 | 1.1287 |
| **8** | *hsa-mir-320e* | 1.9203e-05 | 1.2098e-03 | 2.1526 |
| **9** | *hsa-mir-4488* | 1.0016e-05 | 1.2098e-03 | 1.9790 |
| **10** | *hsa-mir-532* | 1.8218e-05 | 1.2098e-03 | -1.0067 |
| **11** | *hsa-mir-6131* | 1.8842e-05 | 1.2098e-03 | 1.3656 |
| **12** | *hsa-mir-625* | 1.4236e-05 | 1.2098e-03 | 1.0319 |
| **13** | *hsa-miR-19b-3p* | 1.2316e-05 | 1.2098e-03 | -1.2165 |
| **14** | *hsa-miR-200c-3p* | 3.7943e-06 | 1.2098e-03 | 2.0229 |
| **15** | *hsa-miR-3158-3p* | 7.8792e-06 | 1.2098e-03 | -1.3829 |
| **16** | *hsa-miR-320e* | 1.9203e-05 | 1.2098e-03 | 2.1526 |
| **17** | *hsa-miR-4488* | 1.0016e-05 | 1.2098e-03 | 1.9790 |
| **18** | *hsa-miR-532-5p* | 1.8218e-05 | 1.2098e-03 | -1.0067 |
| **19** | *hsa-miR-6131* | 1.8842e-05 | 1.2098e-03 | 1.3656 |
| **20** | *hsa-miR-625-3p* | 1.4236e-05 | 1.2098e-03 | 1.0319 |
| **21** | *hsa-mir-12136* | 2.8697e-05 | 1.6435e-03 | 3.6579 |
| **22** | *hsa-miR-12136* | 2.8697e-05 | 1.6435e-03 | 3.6579 |
| **23** | *hsa-mir-320c-2* | 3.8609e-05 | 2.1151e-03 | 0.9340 |
| **24** | *hsa-mir-140* | 5.3393e-05 | 2.5875e-03 | -0.8661 |
| **25** | *hsa-miR-140-3p* | 5.3393e-05 | 2.5875e-03 | -0.8661 |
| **26** | *hsa-miR-320d* | 4.9671e-05 | 2.5875e-03 | 1.0035 |
| **27** | *hsa-miR-320c* | 5.7759e-05 | 2.6954e-03 | 0.9242 |
| **28** | *hsa-mir-320c-1* | 1.0009e-04 | 4.5039e-03 | 0.9133 |
| **29** | *hsa-mir-106b* | 1.0883e-04 | 4.5073e-03 | -0.6744 |
| **30** | *hsa-mir-3909* | 1.1447e-04 | 4.5073e-03 | -0.8899 |
| **31** | *hsa-miR-106b-3p* | 1.0883e-04 | 4.5073e-03 | -0.6744 |
| **32** | *hsa-miR-3909* | 1.1447e-04 | 4.5073e-03 | -0.8899 |
| **33** | *hsa-mir-320d-1* | 1.5293e-04 | 5.8390e-03 | 0.9235 |
| **34** | *hsa-mir-200b* | 2.1042e-04 | 6.2734e-03 | 1.4628 |
| **35** | *hsa-mir-320b-1* | 1.9974e-04 | 6.2734e-03 | 0.8078 |
| **36** | *hsa-mir-320b-2* | 2.1907e-04 | 6.2734e-03 | 0.7977 |
| **37** | *hsa-mir-941-1* | 2.1569e-04 | 6.2734e-03 | -0.7098 |
| **38** | *hsa-mir-941-2* | 2.1569e-04 | 6.2734e-03 | -0.7098 |
| **39** | *hsa-mir-941-3* | 2.1569e-04 | 6.2734e-03 | -0.7098 |
| **40** | *hsa-mir-941-4* | 2.1569e-04 | 6.2734e-03 | -0.7098 |
| **41** | *hsa-mir-941-5* | 2.1569e-04 | 6.2734e-03 | -0.7098 |
| **42** | *hsa-miR-200b-3p* | 2.1042e-04 | 6.2734e-03 | 1.4628 |
| **43** | *hsa-miR-320b* | 2.0621e-04 | 6.2734e-03 | 0.8028 |
| **44** | *hsa-miR-941* | 1.8253e-04 | 6.2734e-03 | -0.7089 |
| **45** | *hsa-mir-19a* | 2.8693e-04 | 7.5318e-03 | -0.8755 |
| **46** | *hsa-mir-4470* | 2.8579e-04 | 7.5318e-03 | 2.0722 |
| **47** | *hsa-miR-19a-3p* | 2.8693e-04 | 7.5318e-03 | -0.8755 |
| **48** | *hsa-miR-4470* | 2.8579e-04 | 7.5318e-03 | 2.0722 |
| **49** | *hsa-mir-26a-1* | 4.1624e-04 | 1.0284e-02 | 0.7010 |
| **50** | *hsa-mir-26a-2* | 4.1166e-04 | 1.0284e-02 | 0.7023 |

**Supplementary Table S6. Top 50 Differentially Expressed miRNAs Between Stage III/IV OVCA and Control.** Top 50 miRNAs differentially expressed between Stage III/IV OVCA and control samples, selected based on statistical significance (adjusted p-value < 0.05) and biological relevance (log2 fold change > 1).

|  | **miRNA** | **p-value** | **Adjusted p-value** | **LFC** |
| --- | --- | --- | --- | --- |
| **1** | *hsa-miR-10a-3p* | 4.32 × 10⁻⁹ | 4.48 × 10⁻⁶ | 2.545 |
| **2** | *hsa-mir-1228* | 1.73 × 10⁻⁸ | 5.98 × 10⁻⁶ | -4.481 |
| **3** | *hsa-miR-1228-5p* | 1.73 × 10⁻⁸ | 5.98 × 10⁻⁶ | -4.481 |
| **4** | *hsa-mir-320e* | 6.08 × 10⁻⁸ | 1.26 × 10⁻⁵ | 3.169 |
| **5** | *hsa-miR-320e* | 6.08 × 10⁻⁸ | 1.26 × 10⁻⁵ | 3.169 |
| **6** | *hsa-mir-25* | 9.50 × 10⁻⁸ | 1.41 × 10⁻⁵ | -1.590 |
| **7** | *hsa-miR-25-3p* | 9.50 × 10⁻⁸ | 1.41 × 10⁻⁵ | -1.590 |
| **8** | *hsa-mir-320d-2* | 4.79 × 10⁻⁷ | 6.21 × 10⁻⁵ | 2.472 |
| **9** | *hsa-mir-532* | 1.29 × 10⁻⁶ | 1.34 × 10⁻⁴ | -1.332 |
| **10** | *hsa-miR-532-5p* | 1.29 × 10⁻⁶ | 1.34 × 10⁻⁄⁴ | -1.332 |
| **11** | *hsa-miR-320d* | 1.61 × 10⁻⁶ | 1.52 × 10⁻⁴ | 2.248 |
| **12** | *hsa-miR-1255b-5p* | 2.04 × 10⁻⁶ | 1.76 × 10⁻⁴ | -1.863 |
| **13** | *hsa-mir-12136* | 3.34 × 10⁻⁶ | 2.07 × 10⁻⁴ | 3.989 |
| **14** | *hsa-mir-1255b-1* | 2.92 × 10⁻⁶ | 2.07 × 10⁻⁴ | -1.834 |
| **15** | *hsa-mir-1255b-2* | 2.75 × 10⁻⁶ | 2.07 × 10⁻⁴ | -1.841 |
| **16** | *hsa-mir-4488* | 3.60 × 10⁻⁶ | 2.07 × 10⁻⁴ | 3.033 |
| **17** | *hsa-miR-12136* | 3.34 × 10⁻⁶ | 2.07 × 10⁻⁴ | 3.989 |
| **18** | *hsa-miR-4488* | 3.60 × 10⁻⁶ | 2.07 × 10⁻⁴ | 3.033 |
| **19** | *hsa-mir-3158-1* | 5.06 × 10⁻⁶ | 2.38 × 10⁻⁴ | -1.638 |
| **20** | *hsa-mir-3158-2* | 5.06 × 10⁻⁶ | 2.38 × 10⁻⁴ | -1.638 |
| **21** | *hsa-mir-320d-1* | 4.37 × 10⁻⁶ | 2.38 × 10⁻⁴ | 2.097 |
| **22** | *hsa-miR-3158-3p* | 4.91 × 10⁻⁶ | 2.38 × 10⁻⁴ | -1.640 |
| **23** | *hsa-mir-130a* | 6.02 × 10⁻⁶ | 2.60 × 10⁻⁴ | 1.650 |
| **24** | *hsa-miR-130a-3p* | 6.02 × 10⁻⁶ | 2.60 × 10⁻⁴ | 1.650 |
| **25** | *hsa-mir-3182* | 1.38 × 10⁻⁵ | 5.21 × 10⁻⁴ | 2.104 |
| **26** | *hsa-mir-320c-1* | 1.32 × 10⁻⁵ | 5.21 × 10⁻⁴ | 1.962 |
| **27** | *hsa-miR-3182* | 1.38 × 10⁻⁵ | 5.21 × 10⁻⁴ | 2.104 |
| **28** | *hsa-miR-320c* | 1.40 × 10⁻⁵ | 5.21 × 10⁻⁴ | 1.938 |
| **29** | *hsa-mir-320c-2* | 1.57 × 10⁻⁵ | 5.61 × 10⁻⁴ | 1.914 |
| **30** | *hsa-miR-6741-3p* | 1.84 × 10⁻⁵ | 6.35 × 10⁻⁴ | -2.999 |
| **31** | *hsa-miR-144-3p* | 3.10 × 10⁻⁵ | 1.04 × 10⁻³ | 2.215 |
| **32** | *hsa-mir-200c* | 4.90 × 10⁻⁵ | 1.54 × 10⁻³ | 2.521 |
| **33** | *hsa-miR-200c-3p* | 4.90 × 10⁻⁵ | 1.54 × 10⁻³ | 2.521 |
| **34** | *hsa-mir-144* | 1.21 × 10⁻⁴ | 3.47 × 10⁻³ | 2.057 |
| **35** | *hsa-mir-4429* | 1.19 × 10⁻⁴ | 3.47 × 10⁻³ | 1.188 |
| **36** | *hsa-miR-4429* | 1.19 × 10⁻⁴ | 3.47 × 10⁻³ | 1.188 |
| **37** | *hsa-mir-15a* | 1.60 × 10⁻⁴ | 3.95 × 10⁻³ | 1.802 |
| **38** | *hsa-mir-181b-1* | 1.64 × 10⁻⁴ | 3.95 × 10⁻³ | 0.993 |
| **39** | *hsa-mir-181b-2* | 1.67 × 10⁻⁴ | 3.95 × 10⁻³ | 0.987 |
| **40** | *hsa-mir-26a-1* | 1.64 × 10⁻⁴ | 3.95 × 10⁻³ | 0.718 |
| **41** | *hsa-mir-26a-2* | 1.61 × 10⁻⁴ | 3.95 × 10⁻³ | 0.719 |
| **42** | *hsa-miR-15a-5p* | 1.60 × 10⁻⁴ | 3.95 × 10⁻³ | 1.802 |
| **43** | *hsa-miR-181b-5p* | 1.56 × 10⁻⁴ | 3.95 × 10⁻³ | 0.989 |
| **44** | *hsa-miR-26a-5p* | 1.62 × 10⁻⁴ | 3.95 × 10⁻³ | 0.718 |
| **45** | *hsa-mir-1290* | 2.04 × 10⁻⁴ | 4.60 × 10⁻³ | 1.639 |
| **46** | *hsa-miR-1290* | 2.04 × 10⁻⁴ | 4.60 × 10⁻³ | 1.639 |
| **47** | *hsa-mir-1224* | 2.31 × 10⁻⁴ | 5.00 × 10⁻³ | -1.444 |
| **48** | *hsa-miR-1224-5p* | 2.31 × 10⁻⁴ | 5.00 × 10⁻³ | -1.444 |
| **49** | *hsa-mir-320b-1* | 2.51 × 10⁻⁴ | 5.31 × 10⁻³ | 1.351 |
| **50** | *hsa-miR-320b* | 2.63 × 10⁻⁴ | 5.46 × 10⁻³ | 1.341 |

**Supplementary Table S7. Disease Ontology Database Filtered Results for Downstream Target Genes (FDR P < 0.05).** Significant disease ontology terms associated with differentially expressed miRNAs in OVCA. Each entry includes the Disease Ontology (DOID) identifier, associated miRNAs, disease description, adjusted false discovery rate (FDR), and the downstream target genes involved.

| **Disease ID** | **microRNA** | **Description** | **FDR** | **Genes** |
| --- | --- | --- | --- | --- |
| DOID: 2151 | *hsa-miR-19b-3p* | Malignant ovarian surface epithelial-stromal neoplasm | 0.020678509 | PTEN, ESR1, BMPR2, TP53 |
| DOID: 2152 | *hsa-miR-19b-3p* | Ovary epithelial cancer | 0.020678509 | PTEN, ESR1, BMPR2, TP53 |
| DOID: 2394 | *hsa-miR-19b-3p* | Ovarian cancer | 0.024835848 | PTEN, ESR1, BMPR2, TP53 |
| DOID: 3713 | *hsa-miR-19b-3p* | Ovary adenocarcinoma | 0.014887002 | PTEN, TP53 |
| DOID: 4001 | *hsa-miR-19b-3p* | Ovarian carcinoma | 0.020678509 | PTEN, ESR1, BMPR2, TP53 |
| DOID: 5683 | *hsa-miR-19b-3p* | Hereditary breast ovarian cancer | 0.012173141 | PTEN, ESR1, NCOA3, TP53 |
| DOID: 1100 | *hsa-miR-29b-3p* | Ovarian disease | 0.010692033 | VEGFA, ESR1 |
| DOID: 2151 | *hsa-miR-29b-3p* | Malignant ovarian surface epithelial-stromal neoplasm | 0.001309704 | VEGFA, ESR1, TGFB1, PTEN, MYC |
| DOID: 2152 | *hsa-miR-29b-3p* | Ovary epithelial cancer | 0.001309704 | VEGFA, ESR1, TGFB1, PTEN, MYC |
| DOID: 2394 | *hsa-miR-29b-3p* | Ovarian cancer | 0.001697108 | VEGFA, ESR1, TGFB1, PTEN, MYC |
| DOID: 3713 | *hsa-miR-29b-3p* | Ovary adenocarcinoma | 0.005094081 | VEGFA, PTEN |
| DOID: 4001 | *hsa-miR-29b-3p* | Ovarian carcinoma | 0.001309704 | VEGFA, ESR1, TGFB1, PTEN, MYC |
| DOID: 5683 | *hsa-miR-29b-3p* | Hereditary breast ovarian cancer | 9.03471E-05 | VEGFA, ESR1, NCOA3, TGFB1, PTEN, MYC |
| DOID: 2151 | *hsa-miR-30e-5p* | Malignant ovarian surface epithelial-stromal neoplasm | 0.050324906 | TP53 |
| DOID: 2152 | *hsa-miR-30e-5p* | Ovary epithelial cancer | 0.050324906 | TP53 |
| DOID: 3713 | *hsa-miR-30e-5p* | Ovary adenocarcinoma | 0.019307284 | TP53 |
| DOID: 4001 | *hsa-miR-30e-5p* | Ovarian carcinoma | 0.050324906 | TP53 |
| DOID: 5683 | *hsa-miR-30e-5p* | Hereditary breast ovarian cancer | 0.043445352 | TP53 |

**Supplementary Table S8. Differentially Expressed miRNAs Between 14 Diseases, Including OVCA and Control.**

| **Disease vs Control** | **DEMs** | **Upregulated DEMs** | **Downregulated DEMs** |
| --- | --- | --- | --- |
| OVCA | 62 | 6 | 56 |
| Lung Cancer | 28 | 20 | 8 |
| Prostate Cancer | 163 | 59 | 104 |
| Wilms Tumor | 22 | 2 | 20 |
| Melanoma | 270 | 105 | 165 |
| Tumor of Stomach | 44 | 7 | 37 |
| Pancreatic Cancer Ductal | 150 | 106 | 44 |
| Other Pancreatic Tumours | 107 | 73 | 34 |
| Multiple Sclerosis | 199 | 46 | 153 |
| Acute Myocardial Infarction | 229 | 110 | 119 |
| Sarcoidosis | 346 | 218 | 128 |
| Periodontitis | 248 | 62 | 186 |
| Pancreatitis | 117 | 79 | 38 |
| COPD | 135 | 73 | 62 |

**Supplementary Table S9. Differentially Expressed miRNAs Between Stage I/II and Stage III/IV.** miRNAs differentially expressed between Stage I/II OVCA and Stage III/IV OVCA, selected based on statistical significance (adjusted p-value < 0.05) and biological relevance (log2 fold change > 1).

| miRNA ID | baseMean | Log2FC | lfcSE | stat | pvalue | padj |
| --- | --- | --- | --- | --- | --- | --- |
| *hsa-mir-106a* | 77.2895009 | -1.698886 | 0.50254135 | -3.3805894 | 0.00072331 | 0.02366955 |
| *hsa-mir-1228* | 7.63964872 | 2.7968448 | 0.78172569 | 3.57778291 | 0.00034652 | 0.01397916 |
| *hsa-mir-130a* | 163.550877 | -1.3395135 | 0.30676797 | -4.3665363 | 1.2623233770577e-05 | 0.00108904 |
| *hsa-mir-144* | 1569.15652 | -2.0732826 | 0.54835541 | -3.7809103 | 0.00015626 | 0.00926793 |
| *hsa-mir-151b* | 12.8273532 | -1.2510832 | 0.38228679 | -3.27263 | 0.00106552 | 0.03159928 |
| *hsa-mir-15a* | 92.4167684 | -1.9377426 | 0.42328556 | -4.5778613 | 4.69754067746111e-06 | 0.00063685 |
| *hsa-mir-15b* | 193.628829 | -1.1291503 | 0.36997552 | -3.0519595 | 0.00227353 | 0.04448728 |
| *hsa-mir-181b-1* | 291.748811 | -0.742519 | 0.24423007 | -3.0402438 | 0.00236387 | 0.04448728 |
| *hsa-mir-181b-2* | 294.880482 | -0.7487807 | 0.24240794 | -3.0889283 | 0.0020088 | 0.04444125 |
| *hsa-mir-23a* | 794.697729 | -0.9194088 | 0.29406047 | -3.1265977 | 0.00176842 | 0.04303149 |
| *hsa-mir-26b* | 3831.24738 | -1.1979784 | 0.33322482 | -3.5951055 | 0.00032426 | 0.01397916 |
| *hsa-mir-29a* | 584.718686 | -0.9343445 | 0.30826537 | -3.0309744 | 0.00243766 | 0.04448728 |
| *hsa-mir-29b-1* | 14.7604971 | -2.5775884 | 0.48312821 | -5.3352058 | 9.5436246123043e-08 | 4.52844987853839e-05 |
| *hsa-mir-29b-2* | 15.0303447 | -2.429128 | 0.47076081 | -5.1600047 | 2.46943593457901e-07 | 7.81164900638493e-05 |
| *hsa-mir-29c* | 40.3736932 | -1.7293817 | 0.40282759 | -4.2931063 | 1.76190567601865e-05 | 0.00128619 |
| *hsa-mir-30b* | 82.8762074 | -1.7170778 | 0.38457953 | -4.4648185 | 8.01366588346898e-06 | 0.000845 |
| *hsa-mir-30e* | 4290.57364 | -0.9714756 | 0.30829609 | -3.1511123 | 0.0016265 | 0.04171751 |
| *hsa-mir-3182* | 26.9969205 | -1.5479563 | 0.42835924 | -3.6136872 | 0.00030187 | 0.01397916 |
| *hsa-mir-320d-2* | 527.411638 | -1.3829834 | 0.44782708 | -3.0882086 | 0.00201367 | 0.04444125 |
| *hsa-mir-324* | 10.160483 | -2.423973 | 0.68605143 | -3.5332234 | 0.00041053 | 0.01498418 |
| *hsa-mir-374b* | 90.8036412 | -1.7388671 | 0.36250264 | -4.7968397 | 1.61188496772162e-06 | 0.00030594 |
| *hsa-mir-624* | 4.85977466 | -1.6331047 | 0.53363557 | -3.0603371 | 0.00221088 | 0.04448728 |
| *hsa-mir-652* | 447.76429 | -1.0654758 | 0.32773981 | -3.2509807 | 0.00115008 | 0.03210067 |
| *hsa-mir-660* | 209.175002 | -0.980392 | 0.31600744 | -3.1024334 | 0.00191937 | 0.04442632 |
| *hsa-mir-96* | 74.4820346 | -1.4947865 | 0.41840953 | -3.5725441 | 0.00035353 | 0.01397916 |
| *hsa-let-7a-3p* | 376.536947 | -0.5790508 | 0.1937535 | -2.9885954 | 0.00280263 | 0.04925362 |
| *hsa-miR-106a-5p* | 77.2895009 | -1.698886 | 0.50254135 | -3.3805894 | 0.00072331 | 0.02366955 |
| *hsa-miR-106b-5p* | 208.59376 | -1.2413689 | 0.39037282 | -3.1799574 | 0.00147297 | 0.03882905 |
| *hsa-miR-1228-5p* | 7.63964872 | 2.7968448 | 0.78172569 | 3.57778291 | 0.00034652 | 0.01397916 |
| *hsa-miR-130a-3p* | 163.550877 | -1.3395135 | 0.30676797 | -4.3665363 | 1.2623233770577e-05 | 0.00108904 |
| *hsa-miR-142-5p* | 359.828273 | -1.0686961 | 0.35133381 | -3.0418255 | 0.00235148 | 0.04448728 |
| *hsa-miR-144-3p* | 1536.5275 | -2.185911 | 0.57294641 | -3.8152103 | 0.00013607 | 0.00860851 |
| *hsa-miR-151b* | 12.8273532 | -1.2510832 | 0.38228679 | -3.27263 | 0.00106552 | 0.03159928 |
| *hsa-miR-15a-5p* | 92.4167684 | -1.9377426 | 0.42328556 | -4.5778613 | 4.69754067746111e-06 | 0.00063685 |
| *hsa-miR-15b-5p* | 92.1800534 | -1.7860029 | 0.41383447 | -4.3157422 | 1.59067503579671e-05 | 0.00125796 |
| *hsa-miR-181b-5p* | 586.743007 | -0.7447641 | 0.24276329 | -3.0678613 | 0.00215597 | 0.04448728 |
| *hsa-miR-199b-5p* | 66.4802667 | -1.0732376 | 0.32552854 | -3.2969079 | 0.00097756 | 0.03092334 |
| *hsa-miR-223-3p* | 956.75163 | -1.3515877 | 0.39746261 | -3.4005405 | 0.00067253 | 0.0236381 |
| *hsa-miR-23a-3p* | 794.697729 | -0.9194088 | 0.29406047 | -3.1265977 | 0.00176842 | 0.04303149 |
| *hsa-miR-26b-5p* | 3831.24738 | -1.1979784 | 0.33322482 | -3.5951055 | 0.00032426 | 0.01397916 |
| *hsa-miR-29a-3p* | 584.718686 | -0.9343445 | 0.30826537 | -3.0309744 | 0.00243766 | 0.04448728 |
| *hsa-miR-29b-3p* | 28.8548825 | -2.6513407 | 0.48873161 | -5.424942 | 5.79733500423844e-08 | 4.52844987853839e-05 |
| *hsa-miR-29c-3p* | 40.2256814 | -1.7217024 | 0.40859934 | -4.213669 | 2.51255374147915e-05 | 0.00170315 |
| *hsa-miR-29c-5p* | 6.80644787 | -1.3709473 | 0.44621901 | -3.0723642 | 0.0021237 | 0.04448728 |
| *hsa-miR-30b-5p* | 81.9664524 | -1.766145 | 0.39217643 | -4.5034452 | 6.68606161018159e-06 | 0.00079313 |
| *hsa-miR-3182* | 26.9969205 | -1.5479563 | 0.42835924 | -3.6136872 | 0.00030187 | 0.01397916 |
| *hsa-miR-320d* | 1240.70135 | -1.282984 | 0.42704026 | -3.0043631 | 0.00266138 | 0.04765368 |
| *hsa-miR-324-5p* | 10.160483 | -2.423973 | 0.68605143 | -3.5332234 | 0.00041053 | 0.01498418 |
| *hsa-miR-374b-5p* | 90.8036412 | -1.7388671 | 0.36250264 | -4.7968397 | 1.61188496772162e-06 | 0.00030594 |
| *hsa-miR-624-5p* | 4.85977466 | -1.6331047 | 0.53363557 | -3.0603371 | 0.00221088 | 0.04448728 |
| *hsa-miR-628-5p* | 5.85990734 | -1.6493361 | 0.51708229 | -3.1896975 | 0.00142422 | 0.03861665 |
| *hsa-miR-652-3p* | 447.76429 | -1.0654758 | 0.32773981 | -3.2509807 | 0.00115008 | 0.03210067 |
| *hsa-miR-660-5p* | 209.175002 | -0.980392 | 0.31600744 | -3.1024334 | 0.00191937 | 0.04442632 |
| *hsa-miR-96-5p* | 74.4820346 | -1.4947865 | 0.41840953 | -3.5725441 | 0.00035353 | 0.01397916 |

## Supplementary Section S2: Extended Functional Enrichment Analysis

**Supplementary Table S10: Gene Ontology Database Filtered Results for Downstream Target Genes (FDR P < 0.05).** This table shows all the significantly enriched Gene Ontology (GO) biological processes associated with downstream target genes of the differentially expressed miRNAs identified in ovarian cancer. The selection was based on fold enrichment and statistical significance (FDR < 0.05).

| **GO Biological Process Complete** | **REFLIST (20589)** | **Filtered Gene List (8)** | **Expected** | **Over/**  **Under** | **Fold Enrichment** | **Raw P-Value** | **FDR** |
| --- | --- | --- | --- | --- | --- | --- | --- |
| Positive regulation of cell proliferation by VEGF-activated platelet-derived growth factor receptor signaling pathway (GO:0038091) | 3 | 1 | 0 | + | > 100 | 1.55E-03 | 4.46E-02 |
| Positive regulation of miRNA maturation (GO:1903800) | 12 | 2 | 0 | + | > 100 | 1.20E-05 | 1.25E-03 |
| Positive regulation of SMAD protein signal transduction (GO:0060391) | 19 | 2 | 0.01 | + | > 100 | 2.76E-05 | 2.24E-03 |
| Positive regulation of miRNA-mediated gene silencing (GO:2000637) | 24 | 2 | 0.01 | + | > 100 | 4.27E-05 | 2.99E-03 |
| Positive regulation of post-transcriptional gene silencing (GO:0060148) | 25 | 2 | 0.01 | + | > 100 | 4.61E-05 | 3.13E-03 |
| Positive regulation of post-transcriptional gene silencing by RNA (GO:1900370) | 25 | 2 | 0.01 | + | > 100 | 4.61E-05 | 3.12E-03 |
| Positive regulation of miRNA transcription (GO:1902895) | 47 | 3 | 0.02 | + | > 100 | 7.47E-07 | 1.65E-04 |
| Positive regulation of miRNA metabolic process (GO:2000630) | 53 | 3 | 0.02 | + | > 100 | 1.06E-06 | 2.07E-04 |
| Positive regulation of fibroblast proliferation (GO:0048146) | 54 | 3 | 0.02 | + | > 100 | 1.11E-06 | 2.13E-04 |
| Positive regulation of epithelial cell proliferation (GO:0050679) | 209 | 4 | 0.08 | + | 49.26 | 7.53E-07 | 1.64E-04 |
| Positive regulation of cell population proliferation (GO:0008284) | 949 | 6 | 0.37 | + | 16.27 | 2.53E-07 | 9.67E-05 |

**Supplementary Table S11: PANTHER Pathway Database Results for Downstream Target Genes (FDR P < 0.05)**

| **PANTHER Pathways** | **REFLIST**  **(20589)** | **Filtered Gene List (8)** | **Expected** | **Over/**  **Under** | **Fold Enrichment** | **Raw P-Value** | **FDR** |
| --- | --- | --- | --- | --- | --- | --- | --- |
| p53 pathway feedback loops 2 (P04398) | 51 | 3 | 0.02 | + | > 100 | 9.45E-07 | 1.51E-04 |
| p53 pathway (P00059) | 89 | 2 | 0.03 | + | 57.83 | 5.31E-04 | 2.13E-02 |
| TGF-beta signaling pathway (P00052) | 102 | 2 | 0.04 | + | 50.46 | 6.93E-04 | 2.22E-02 |
| Gonadotropin-releasing hormone receptor pathway (P06664) | 237 | 3 | 0.09 | + | 32.58 | 8.38E-05 | 4.47E-03 |
| Unclassified (UNCLASSIFIED) | 17971 | 1 | 6.98 | - | 0.14 | 3.86E-06 | 3.08E-04 |

## Supplementary Section S3: Prediction Model Details

**Supplementary Table S12: Additional Classifier Parameters for the RandomForest Model**

| **Parameter** | **Value** |
| --- | --- |
| **Best Classifier** | weka.classifiers.trees.RandomForest |
| **Arguments** | [-I, 10, -K, 0, -depth, 0] |
| **Metric** | ErrorRate |
| **Estimated Error Rate** | 0.0417 |
| **Kappa Statistic** | 0.8735 |
| **Mean Absolute Error** | 0.1375 |
| **Root Mean Squared Error** | 0.2227 |
| **Relative Absolute Error** | 31.4% |
| **Root Relative Squared Error** | 47.6% |
| **Total Number of Instances** | 48 |
