# Supplementary material for: A circulating three-miRNA panel (hsa-miR-29b-3p, hsa-miR-19b-3p, hsa-miR-30e-5p) for early-stage ovarian cancer detection: a machine-learning bioinformatics approach
Source: Front Genet. 2026 Aug 12;17:1827636. doi: 10.3389/fgene.2026.1827636 (PMC13506143; doi:10.3389/fgene.2026.1827636)
Supplement: Supplementary file 2 [file DataSheet1.zip › Supplementary Figures.docx]

**Supplementary Figure S1. Multi-panel volcano plot for OVCA vs different cancer types.** From top left to top right: OVCA vs BRCA, OVCA vs COAD, OVCA vs CESC and OVCA vs UCEC.

**Supplementary Figure S2.** **Heatmap of Top Differentially Expressed miRNAs Between OVCA and BRCA.**

**Supplementary Figure S3. Heatmap for OVCA vs CESC.**

**Supplementary Figure S4. Heatmap for OVCA vs UCEC.**

**Supplementary Figure S5. Heatmap for OVCA vs COAD.**

**Supplementary Figure S6. Volcano plot for different stages of OVCA vs. control.**

**Supplementary Figure S7. Heatmap for Benign OVCA vs. Control.**

**Supplementary Figure S8**. **Heatmap for Borderline OVCA vs Control.**

**Supplementary Figure S9. Heatmap for Stage I/II OVCA vs Control.**

**Supplementary Figure S10. Heatmap for Stage III/IV OVCA vs Control.**

**Supplementary Figure S11. Venn diagram for stage-specific DEMs.** This Venn diagram illustrates the overlap of differentially expressed miRNAs specific to early-stage and late-stage ovarian cancer, emphasizing stage-specific biomarkers. There are 45 stage III/IV-specific miRNAs and 29 miRNAs in common between early and late-stage OVCA.

**Supplementary Figure S12. Comparative DEMs across diseases.**

**Supplementary Figure S13. Volcano Plot of Differentially Expressed miRNAs Between OVCA and Control Samples.**

**Supplementary Figure S14. Heatmap of Top Differentially Expressed miRNAs Between OVCA and Control Samples.**
